# Utility and Limitations of Fine-Needle Aspiration Cytology in the Diagnosis of Lymphadenopathy

**DOI:** 10.3390/diagnostics13040728

**Published:** 2023-02-14

**Authors:** Hwa Jeong Ha, Jeeyong Lee, Da Yeon Kim, Jung-Soon Kim, Myung-Soon Shin, Insup Noh, Jae Soo Koh, Eun Ju Kim, Seung-Sook Lee

**Affiliations:** 1 Department of Pathology, Korea Cancer Center Hospital, Korea Institute of Radiological & Medical Sciences, Seoul 01812, Republic of Korea; 2 Convergence Institute of Biomedical Engineering and Biomaterials, Seoul National University of Science and Technology, Seoul 01811, Republic of Korea; 3 Division of Radiation Biomedical Research, Korea Institute of Radiological & Medical Sciences, Seoul 0182, Republic of Korea; 4 Radiological & Medico-Oncological Sciences, University of Science & Technology, Daejeon 34113, Republic of Korea; 5 Department of Chemical and Biomolecular Engineering, Seoul National University of Science and Technology, Seoul 01811, Republic of Korea; 6 Institute for Molecular Bioscience, The University of Queensland, Carmody Rd, St Lucia, QLD 4072, Australia

**Keywords:** fine-needle aspiration cytology (FNAC), histopathology, lymphadenopathy

## Abstract

Background: Fine needle aspiration cytology (FNAC) is a valuable tool for evaluating lymphadenopathy. The purpose of this study was to assess the reliability and effectiveness of FNAC in the diagnosis of lymphadenopathy. Methods: Cytological characteristics were evaluated in 432 patients who underwent lymph node FNAC and follow-up biopsy at the Korea Cancer Center Hospital from January 2015 to December 2019. Results: Fifteen (3.5%) of the four hundred and thirty-two patients were diagnosed as inadequate by FNAC, with five (33.3%) of these diagnosed as metastatic carcinoma on histological examination. Of the 432 patients, 155 (35.9%) were diagnosed as benign by FNAC, with seven (4.5%) of these diagnosed histologically as metastatic carcinoma. A review of the FNAC slides, however, showed no evidence of cancer cells, suggesting that the negative results may have been due to FNAC sampling errors. An additional five samples regarded as benign on FNAC were diagnosed as non-Hodgkin lymphoma (NHL) by histological examination. Of the 432 patients, 223 (51.6%) were cytologically diagnosed as malignant, with 20 (9.0%) of these diagnosed as tissue insufficient for diagnosis (TIFD) or benign on histological examination. A review of the FNAC slides of these 20 patients, however, showed that 17 (85.0%) were positive for malignant cells. The sensitivity, specificity, positive predictive value (PPV), negative predictive values (NPV), and accuracy of FNAC were 97.8%, 97.5%, 98.7%, 96.0%, and 97.7%, respectively. Conclusions: Preoperative FNAC was safe, practical, and effective in the early diagnosis of lymphadenopathy. This method, however, had limitations in some diagnoses, suggesting that additional attempts may be required according to the clinical situation.

## 1. Introduction

Lymphadenopathy is a frequent occurrence in clinical settings [1]. The diagnosis of enlarged lymph nodes solely by clinical judgment may be challenging, however, as the clinical symptoms of lymphadenopathy may be similar to those of other conditions. Although tissue biopsy is the most reliable diagnostic method of determining the cause of lymph node enlargement, this method is invasive and costly, limiting its application.

The limitations of tissue biopsy have led to the use of fine needle aspiration cytology (FNAC) as an initial diagnostic method at many institutions. However, it was not universally accepted, primarily due to a lack of rules and a classification for cytopathological diagnoses. For lymph node fine needle aspiration cytology (LN-FNAC), a group of cytopathologists has created a performance, category, and reporting system, the so-called Sydney system [2]. A better interdisciplinary understanding of the outcomes of this technique and a broader acceptance and utilization of LN-FNAC are both possible outcomes of this system [3,4,5]. The advantages of FNAC of lymph nodes in the initial diagnosis and the management of patients with lymphadenopathy include the early availability of results, simplicity, and minimal trauma with fewer complications [6].

FNAC has shown high accuracy in diagnosing reactive lymphoid hyperplasia, infectious diseases, granulomatous lymphadenitis, and metastatic malignancy [2]. The diagnostic accuracy of FNAC, however, may be lower in patients with primary lymphoproliferative disorders [7]. Early reports suggested that FNAC produced high false negative rates in patients with Hodgkin lymphoma (HL) and low-grade non-Hodgkin lymphoma. More recent studies have indicated that FNAC can accurately diagnose lymphoma in 85–90% of patients, particularly when ancillary techniques complement morphological assessment [8,9]. Ancillary techniques, such as immunohistochemistry (IHC), can overcome these difficulties and support the interpretation of cytological diagnoses [10,11]. However, the role of FNAC in the initial diagnosis and subclassification of primary lymphoid malignancy remains unclear. A cytological diagnosis of lymphoma on FNAC is often followed by tissue biopsy [12].

Diagnosing tumors that have metastasized to the lymph nodes on cytological smears is crucial, as it may be the sole indication for searching for the primary tumor, especially in patients with occult carcinoma [13]. In most of these patients, however, the primary tumor has been identified clinically, with FNAC used widely for patient follow-up. Although most metastatic carcinomas can be identified solely by their cytomorphological characteristics, the features of different tumors may overlap, limiting the precise diagnosis of the primary tumor [14].

The present study assessed the reliability and validity of FNAC by comparing the cytological and histological diagnoses of 432 patients with lymph node enlargement. It was intended to help establish an appropriate treatment plan for future patients by increasing the diagnosis rate of malignant tumors and reducing false-negative and false-positive rates.

## 2. Materials and Methods

### 2.1. Study Design

A total of 2517 lymph node FNACs were retrieved and evaluated retrospectively between January 2015 and December 2019. Subsequent biopsy specimens were available in 432 patients for the confirmation of the diagnosis. These 432 samples histologically diagnosed as tissue insufficient diagnosis (TIFD) (*n* = 39), benign (*n* = 155), and malignant (*n* = 238) were reviewed and compared with the corresponding cytologic diagnoses.

As for the diagnostic results, cytologic smears identified as sampling errors, overdiagnosis, and underdiagnosis were reviewed and diagnostic sensitivity, specificity, and accuracy were calculated. Cytologic diagnoses were classified as inadequate/non-diagnostic, benign, atypical, suspicious, and malignant. Samples suspected of malignancy were included in those diagnosed as malignant. Slides were reviewed if cytological and histological diagnoses did not match, with the reason for non-matching classified as a sampling or interpretative error.

This study was approved by the Institutional Review Board of Korea Cancer Center Hospital, which waived the requirement for patient informed consent (IRB FILE No. 2020-11-003-003).

### 2.2. Methods

#### Obtaining Specimens and Ancillary Tests

In general, cytology was performed on specimens using ultra-sonography guided fine needle aspiration (USG-FNA) by radiologists at the department of radiology. However, the FNAC of mediastinal lymph nodes was performed using endobronchial ultrasound-guided (EBUS) transbronchial FNAC by pulmonologists. It uses sound waves to help find a nodule or other abnormalities inside the lymph node. This method is rapid, inexpensive, does not expose the patient to ionizing radiation, is less invasive than surgical biopsy, and leaves little to no scarring. It is carried out with a 10-mL plastic syringe and a standard 23-gauge needle without aspiration equipment. The target node localizes in the US monitor’s center using the US probe. The collected material was smeared on four glass slides and quickly fixed in 95% ethyl alcohol. The syringe was rinsed with normal saline for any remaining material to be used for making cell blocks. The FNAC smear slides were stained using an automated Papanicolaou Stainer (Thermo Scientific, Walldorf, Germany) and cell blocks were cut into 4-μm sections and stained with Hematoxylin and Eosin. When differential diagnosis was required, IHC was performed using a cell block with a Bond-III automatic slide Stainer (Leica Biosystems Melbourne Pty., Ltd. VIC, Melbourne, Australia).

We had cell blocks in 273 cases out of 432 LN-FNAC. However, not all the CB slides provided findings helpful for diagnosis. Of the 20 cases diagnosed as NHL by FNAC, 4 cases were diagnosed with cell block, and IHC was performed for the four cases that cell block was available. Regarding ancillary tests, immunohistochemistry was performed on cell block from 27 patients of total 432, and EGFR mutation tests were carried out for three cases (total IHC; 27/432, non-Hodgkin lymphoma IHC; 4/20).

### 2.3. Statistical Analysis

The sensitivity, specificity, positive predictive value (PPV), negative predictive value (NPV), and diagnostic accuracy of lymph node FNAC were assessed.

In FNAC diagnosis, we utilized two forms of analysis. In the group I, malignant and suspicious malignancies were classified as True Positive (TP), benign as True Negative (TN), false positive as False Positive (FP), and false negative as False Negative (FN). Inadequate/non-diagnostic and atypical cells were excluded from this analysis. In the group II, TP included malignant, suspicious malignancy, and atypical. In this second group, inadequate/non-diagnostic were excluded from analysis. Sensitivity, specificity, positive predictive value (PPV), negative predictive values (NPV), and accuracy were calculated as below.
SensitivityTP/(TP + FN)SpecificityTN/(TN + FP)Positive predictive value (PPV)TP/(TP + FP)Negative predictive values (NPV)TN/(TN + FN)Accuracy(TP + TN)/(TP + FP + TN + FN)

## 3. Results

### 3.1. Analysis of Lymphadenopathy Using Ultra-Sonography Guided FNAC

Samples from 432 patients who underwent biopsy were reviewed: 247 were male and 185 were female, with their ages ranging from 8 to 89 years (mean age 58.2 years). Of these patients, 34 were aged <30 years, 96 were aged 30 to 50 years, and 302 were aged >50 years. The mean size of the enlarged lymph nodes was 1.9 cm (range, 0.2–5.0 cm); of these lymph nodes, 265 were <2.0 cm, 120 were 2.0 to 3.0 cm, 47 were >3.0 cm, and 47 were >3.0 cm, with the largest being 5.0 cm. The evaluation of their location showed that 257 LNs were cervical, 59 were supraclavicular, 86 were mediastinal, 22 were axillary, and 8 were inguinal (Table 1).

### 3.2. Comparison of Initial Cytologic and Histologic Diagnoses

Histologic diagnoses were compared with cytological diagnoses by FNAC in the 432 patients (Table 2) [15]. Of the fifteen patients diagnosed as inadequate/non-diagnostic on FNAC, three were diagnosed histologically with TIFD, seven with benign lesions, and five with metastases. Of the 155 patients diagnosed as having benign lesions on FNAC, 24 were diagnosed histologically with TIFD, 119 with benign lesions, 7 with metastatic carcinomas, and 5 with NHL. Of the thirty-nine samples diagnosed as having atypical cells on FNAC, five were diagnosed histologically with TIFD, sixteen with benign lesions, twelve with metastatic carcinoma, and six with NHL. Of the seven samples diagnosed as suspicious for malignancy by FNAC, three were diagnosed histologically with benign lesions, three with metastatic carcinomas, and one with NHL. Of the 192 patients diagnosed with metastatic carcinoma by FNAC, 7 were histologically diagnosed as TIFD, 10 as benign, 172 as metastatic carcinoma, and 3 as NHL. Of the 35 patients diagnosed histologically with NHL, 20 were correctly diagnosed as NHL by FNAC. All four patients diagnosed histologically with HL were cytologically diagnosed as HL.

Out of the 172 cases of metastatic carcinoma, lung cancer (58 cases) was the primary site, followed by thyroid cancer (50 cases). The distribution of the primary sites of the metastatic tumors in 172 LN-FNAC is summarized in Table 3. In addition, the primary sites of metastasis were breast in eighteen cases and the oral cavity in nine cases. Skin and tonsils were the primary sites in five cases each, the pharynx in four cases, and the esophagus, ovary, and bladder were each three cases. The salivary gland, larynx, stomach, and rectum were the primary sites in 2 cases each; the liver, kidney, and cervix were the primary sites in one case each; and three cases had primary sites of unknown origin.

### 3.3. Sampling Errors of FNAC

Of the fifteen patients diagnosed cytologically as inadequate/non-diagnostic (Table 2), three were diagnosed histologically with TIFD, seven as benign, and five as metastatic. Of the latter five patients, two were diagnosed with ductal carcinoma and one each with adenocarcinoma, squamous cell carcinoma, and papillary thyroid carcinoma.

Of the 155 patients diagnosed cytologically as benign, 7 were histologically diagnosed as having metastatic carcinoma, but no cancer cells were observed in the cytology slides. Therefore, 22 patients (15 diagnosed cytologically as inadequate/non-diagnostic and 7 diagnosed cytologically as benign) could not be accurately diagnosed due to errors in FNAC sampling (Table 4).

### 3.4. Sampling Errors of Biopsy

In this series, 39 inadequate tissue specimens were all from needle biopsy. Interestingly, most of the 39 inadequate biopsies (35/39) were derived from the EBUS-transbronchial needle biopsy for mediastinal lymph nodes. The tissues were too small to be properly evaluated and/or were non-lymphoid tissues.

**Figure 1:**
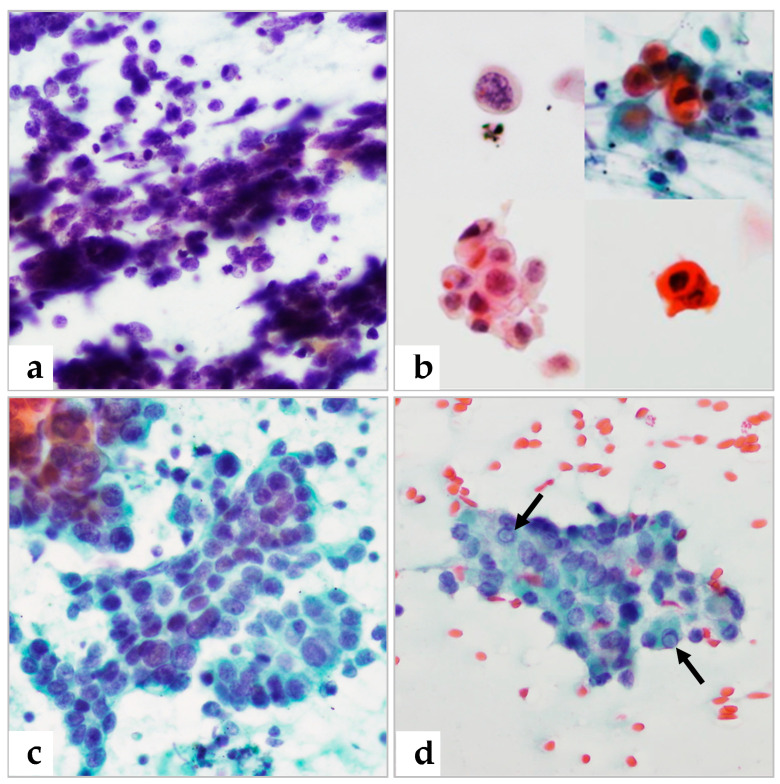
Presence of cancer cells on FNAC slides coupled with absence of tumor cells on biopsy slides due to tissue sampling errors. (**a**) A metastatic small cell carcinoma exhibiting a crush artifact and apoptosis of individual cells. (**b**) A metastatic keratinizing squamous cell carcinoma. Nuclei are hyperchromatic with irregular membranes or pyknotic with dense orangeophilic cytoplasm (composite figure of four different areas). (**c**) Syncytial tissue fragments of adenocarcinoma cells. (**d**) Metastatic papillary thyroid carcinoma showing intranuclear cytoplasmic inclusions (arrows). (**a**–**d**): Papanicolaou stain, 400× magnification.

Of the 192 patients cytologically diagnosed with metastasis, 7 were diagnosed with TIFD and 10 with benign lesions on retrospective biopsy (Table 2). All seven patients with TIFD and nine of the ten with benign lesions were confirmed as having malignancy on cytologic examination (Figure 1), with one patient classified as benign, displaying reactive hyperplasia on histological diagnosis. Thereby, forty-nine patients showed sampling error in tissue specimen, with thirty-nine histologically diagnosed with TIFD (including the seven described above), one with malignant melanoma, and nine with metastasis (Table 5).

### 3.5. Cytologic Over-Diagnoses

FNAC yielded false positive results in three patients. Two were overdiagnosed as suspicious for malignancy and the last as having metastatic papillary thyroid carcinoma. Histological diagnoses showed that one of these patients had a foreign body granuloma and two had reactive hyperplasia.

The patient diagnosed with foreign body granuloma on biopsy following FNAC had a previous history of squamous cell carcinoma. FNAC showed many keratinized squamous cells, which might mislead to the suggestion of malignancy. Another patient diagnosed as suspicious for lymphoma by FNAC was confirmed to have reactive hyperplasia by IHC on a follow-up biopsy. In the other patient, some benign follicular cells were misinterpreted as metastatic papillary thyroid carcinoma. All three cases were overdiagnosed cytologically.

### 3.6. Cytologic Under-Diagnoses

**Figure 2:**
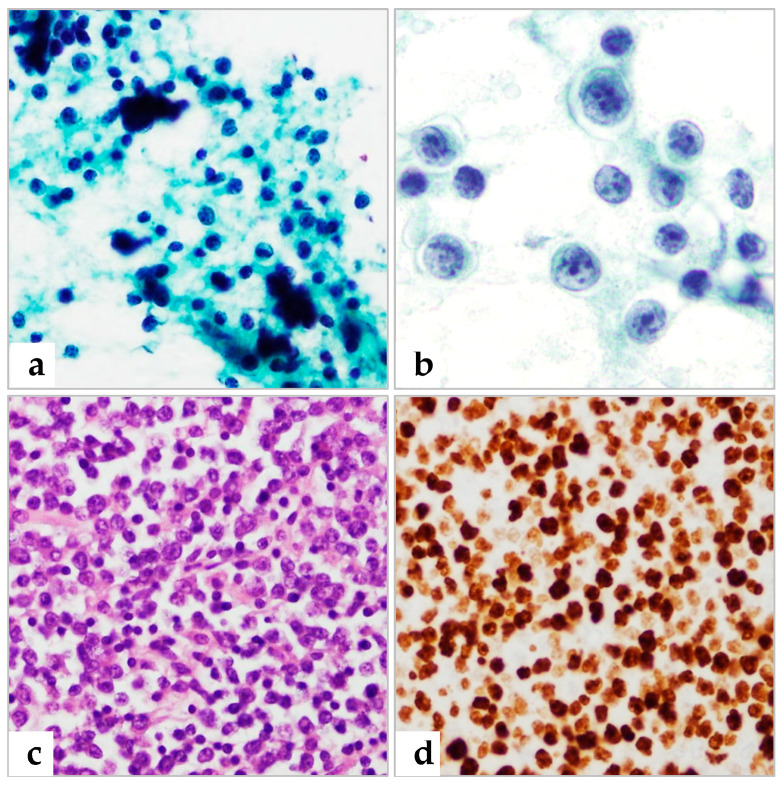
A patient erroneously diagnosed with reactive hyperplasia by FNAC was histologically proven to have diffuse large B cell lymphoma (DLBCL). (**a**,**b**) Pap smear showing a mixture of large and smaller lymphoid cells (**a**) and atypical lymphoid large cells with vesicular chromatin and prominent nucleoli (**b**). (**c**) Histologic section of DLBCL (H&E). (**d**) Ki-67(90%) in IHC. (**a**) 400×; (**b**) 1000×; (**c**,**d**) 400× magnification.

**Figure 3:**
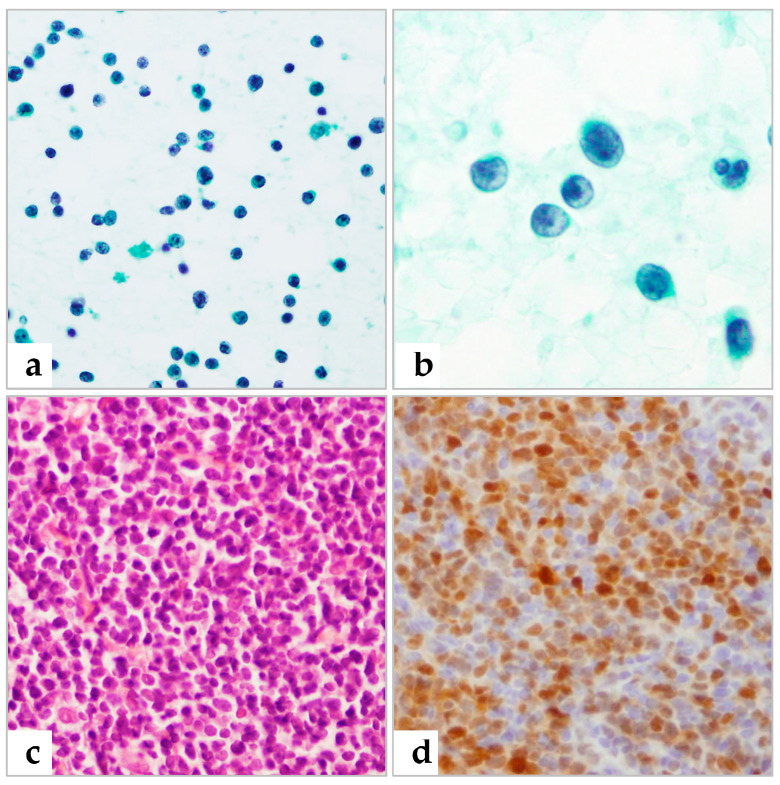
A patient diagnosed with reactive hyperplasia by FNAC was histologically diagnosed with mantle cell lymphoma. (**a**,**b**) Pap staining showing small to intermediate-sized lymphoid cells with finely stippled chromatin (**a**) and monomorphic population of small-cleaved cells. (**c**) Histologic section of mantle cell lymphoma (H&E). (**d**) Positive IHC staining for cyclin D1. (**a**) 400×; (**b**) 1000× (**c**,**d**); 400× magnification.

**Figure 4:**
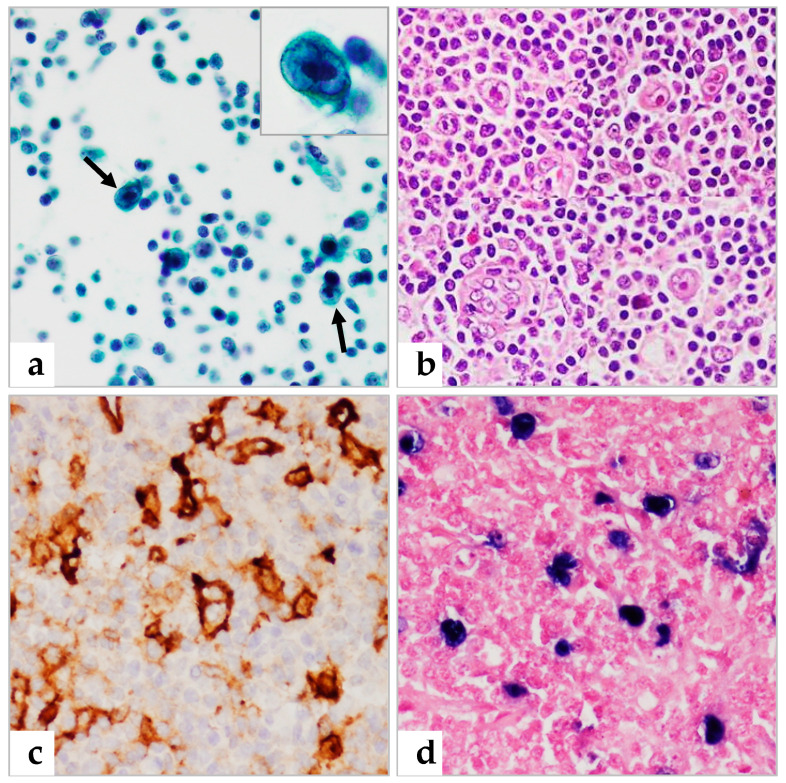
Classical Hodgkin lymphoma. (**a**) Pap smear showing atypical cells (arrows) at lower magnification, with the inset showing a Reed–Sternberg cell. (**b**) Histologic section of Hodgkin lymphoma showing binucleated and multinucleated cells with prominent macronuclei (H&E). (**c**,**d**) IHC positivity for CD30 (**c**) and in situ hybridization for EBV (**d**). (**a**) 400×, insert: 1000×; (**b**–**d**) 400× magnification.

Of the 35 patients with histologically proven NHL (Table 2), 20 were diagnosed with NHL in FNAC prior to biopsy, one was suspicious for lymphoma, and three were erroneously diagnosed with metastatic carcinoma. Six patients were underdiagnosed with atypical lymphoid cells and five were misdiagnosed with reactive hyperplasia (Figure 2 and Figure 3). The five patients having false negative reactive hyperplasia in FNAC were confirmed to NHL, subsequently diagnosed as follicular lymphoma grade 1, mantle cell lymphoma, EBV-positive diffuse large B-cell lymphoma, and two with diffuse large B-cell lymphoma, respectively. These false negatives were partly attributed to interpretation errors, as well as to disease characteristics, making this an error of interpretation during FNAC diagnosis [7,16]. All four patients with histologically proven HL were cytologically diagnosed with HL (Figure 4).

### 3.7. Limitations of Cytological and Histological Diagnoses

In our study, sampling errors and interpretation errors were the main causes of diagnostic limitations. After excluding thirty-nine lymph nodes found to be “atypical” on FNAC, three of the remaining three hundred and ninety-three samples were overdiagnosed, five were underdiagnosed (Table 6), and twenty-two were sampling errors.

Based on these findings, FNAC had the sensitivity, specificity, positive predictive value (PPV), negative predictive values (NPV), and accuracy of 97.8%, 97.5%, 98.7%, 96.0%, and 97.7%, respectively, in group I. In group II, diagnostic accuracy was still high, though lower than group I (Table 7).

## 4. Discussion

FNAC has been used as a primary diagnostic method for lymphadenopathy. In general, to achieve accurate FNAC-based diagnoses, the quality and processing of the cytologic specimen, including its collection, smearing, fixation, and staining, must be reasonable [17,18,19]. In addition, the rapid onset evaluation (ROSE) and ancillary techniques are also important to make an accurate diagnosis [9].

FNAC has been known to accurately diagnose lymphoid hyperplasia, granulomatous lymphadenitis, infectious disease, and metastatic tumors and to be suitable for rapidly monitoring the effects of treatment without the need for an excisional biopsy [20]. When accompanied by clinical and radiographic evidence, FNAC is a diagnostic technique that can prevent unnecessary surgery and accurately identify benign and malignant tumors [21]. In this study, fifteen (3.5%) of the samples showed inadequate specimens with few cellular components, five of the fifteen samples diagnosed as inadequate/non-diagnostic by FNAC were histologically diagnosed as metastatic carcinoma. In contrast, 39 (9.0%) were histologically diagnosed with TIFD, with 7 (18.0%) of these 39 patients diagnosed with metastatic cancer by FNAC. Based on these results, it can be seen that FNAC and biopsy are complementary.

Of the 432 patients analyzed by FNAC, a diagnostic discrepancy of 2.0% was lower than the 9% to 20% previously reported [22,23]. Excluding the 39 cases of atypical cells, there was a false negative rate of 1.3% and a false positive rate of 0.8%. However, our study design, which includes only cases with subsequent histology, may bias the interpretation of the results. There are limitations such as the possible over/under-estimation of the risk of malignancy of some categories, as well as the under-reporting of the LN-FNACs of diseases that were systematically not followed by tissue biopsy (e.g., benign reactive cases but also metastases of a known primary site). False negatives in FNAC may be reduced by selecting a solid site of the nodule and by aspirating multiple sites during the sample collection [24,25]. Collecting sufficient specimens from the correct lesions with more careful inspection is necessary to increase diagnostic accuracy.

In the present study, the most frequently diagnosed lesion was metastatic carcinoma, for which FNAC had a diagnostic accuracy of 96.5% (192/199 patients, including those suspicious for metastatic carcinoma). This was in good agreement with the cytological diagnosis rate reported in the literature for metastatic carcinoma in lymph nodes, which was 90–100% [26,27]. Under certain circumstances, however, the cytological analysis of lymph node cellular samples obtained by FNAC and smears on slides does not provide the same information as conventional histological approaches because of errors in collection and interpretation.

Regarding metastatic carcinoma, the cytologic diagnosis of FNAC showed a very high correlation with histological findings in this study. When cases diagnosed as metastatic cancer by FNAC but diagnosed as TIFD or benign by histological examination were reviewed, definite tumor cells were found in the FNAC specimen. This suggests that FNAC can sometimes compensate for the limitations of needle biopsy.

In this study, the primary sites of metastatic carcinoma were as follows: lung, thyroid, breast, and others. In addition, various primary sites are summarized in Table 3. As the FNAC sites are mainly in the cervical, supraclavicular, and mediastinal areas (Table 1), lung or thyroid are the most common primary sites.

Among the 199 cases of metastatic carcinoma in histological diagnosis, 7 cases diagnosed as benign in FNAC correspond to a kind of sampling error in which no tumor cells are observed. In addition, the 12 cases diagnosed as atypical by FNAC were difficult to diagnose definitively due to the low number of tumor cells.

As health screenings, such as ultrasonographic examination, have become more common, the detection rate and incidence of thyroid cancer has significantly increased. In this regard, FNAC is highly useful not only in the thyroid gland but also in cervical lymphadenopathy. Thyroid cancer is a tumor with frequent recurrence of cervical lymph node metastases after surgery. Therefore, if cervical lymphadenopathy is detected during the follow-up process, the diagnosis can be easily approached with FNAC. FNAC has the advantage of reaching a diagnosis with reduced patient burden. In addition, even if a small number of metastatic cancer cells are found in the FNAC smear of a thyroid cancer patient, diagnosis can be easily made in most cases along with the past history.

Lung cancer is also a common cancer, and transbronchial lymph node aspiration cytology has recently been performed to evaluate the lymph node status. One additional benefit of LN-FNAC in metastatic lesions is that tissue can be obtained for prognostic-predictive biomarkers. In lung metastases, for example, we can assess the status of EGFR, ALK, ROS, MET, etc., without ever performing a tissue biopsy but only with LN-FNAC [28]. This is also useful for identifying metastasis in patient follow-up. FNAC is essential for the detection of metastatic cancer in the patients with lymphadenopathy and is a good diagnostic method with many advantages in terms of cost–benefit.

FNAC diagnosis is highly accurate and easy to use, so it is expected to be actively used in the future. Moreover, as accurate sampling becomes easier through USG-FNA, its usefulness is further increasing. It would be helpful for patients if we recognize the diagnostic accuracy, usefulness, and limitations of metastatic carcinoma diagnosis in FNAC and referred to it for diagnosis [29].

In particular, the most challenging aspect of lymph node FNAC is that it may not be possible to distinguish between malignant lymphoid lesions and reactive hyperplasia because the actual structure of the lymph node is not seen [30]. In general, lymphomas show the monoplastic proliferation of cancer cells. Nevertheless, the diagnosis rate is lower than that of other lymph node lesions because the correct lesion may not have been aspirated if the lymphoma originates from the lymph node or is mixed with different types of cells [31]. There is a risk of potential cytological misdiagnosis, particularly in lymphomas with an admixed cell pattern (false negatives) or reactive proliferation with atypical cells (false positives). High-grade lymphomas and HLs may show cytomorphological abnormalities evident on aspiration specimens but reactive proliferation is often characterized by a polymorphic cell pattern [32]. High-grade lymphomas, such as diffuse large B-cell lymphoma (DLBCL), usually show a monomorphous smear pattern of medium to large lymphoid cells, which may lead to properly diagnosing lymphoid malignancy on smears. On the other hand, low-grade NHLs, such as follicular lymphomas grades 1 and 2, with minor cytomorphological atypia, remain highly challenging to identify cytologically and are frequently misdiagnosed as reactive lymphoid hyperplasia [33,34]. Difficulties distinguishing between follicular lymphoma and reactive hyperplasia were reported to be mainly due to the large number of small lymphocytes, including histiocytes, that are aspirated with neoplastic cells in follicular lymphoma [35].

In the present study, the rate of misdiagnosis was high between reactive hyperplasia of lymph nodes and malignant lymphoma. A sample suspected of being lymphoma on FNAC was found on histological examination to be reactive hyperplasia. Conversely, five patients diagnosed with reactive hyperplasia in FNAC were proven to lymphoma on histological examination. Three of the five patients were diagnosed with diffuse large B-cell lymphoma and one each with follicular lymphoma and mantle cell lymphoma. Among them, follicular lymphoma and mantle cell lymphoma are known as diagnostic challenging in FNAC. However, the diagnostic error in the three cases of DLBCL in our series seems to be attributed to interpretation errors and suboptimal preparation.

Interestingly, four out of four biopsy-proven HLs in this study were correctly diagnosed by FNAC. FNAC smears in the four HLs revealed characteristic Hodgkin cells in the background of small lymphocytes, which lead to the correct diagnosis. HL has a broad spectrum of histology as well as cytology. Therefore, it is not surprising that the differential diagnosis of HL is comprehensive and includes benign and neoplastic processes. HL must be distinguished from reactive inflammatory processes due to their polymorphic appearance [36,37].

The reactive hyperplasia of lymph nodes was the most common lesion; however, the cytological findings of reactive hyperplasia are challenging [38,39]. Because of these limitations of FNAC, there have been certain studies showing that incisional biopsy and other auxiliary diagnostic methods are very helpful for diagnosis [40,41]. Although efforts have been made to use IHC diagnostically, even in FNAC [42], it may not always be helpful. Therefore, excisional biopsy has therefore been recommended to support the primary cytological diagnosis of lymphoma. Regarding lymphoma diagnosis, FNAC is not sufficient to make an accurate diagnosis because structural pattern and IHC study is important for the lymphoma diagnosis. Recognizing these limitations, if lymphoma is clinically suspected, a biopsy (particularly an excisional biopsy) should be recommended. Additionally, FNAC is useful to allow the sampling of multiple sites for staging in order to exclude other unrelated causes of lymphadenopathy and to obtain samples from surgically inaccessible areas or medically unfit patients [43,44,45].

Histology is regarded as the definitive method for diagnosing lymph nodes. Incisional biopsy should be considered when cytology is not sufficiently accurate or when FNAC results differ from clinical findings. In this study, however, FNAC showed a high diagnostic accuracy of 97.6%, suggesting that FNAC should be performed first. Although the results of FNAC may not always match the clinical results, FNAC is a valuable and essential test method because its high diagnostic accuracy suggests that diagnosis and treatment will not be altered, even if a biopsy is later performed [46].

## 5. Conclusions

In summary, this study showed that FNAC had an overall diagnostic accuracy of 97.6%. The misdiagnosis of the remaining patients was due to sampling error or misinterpretation, especially in lymphoma diagnosis. Nevertheless, the FNAC of lymph nodes has become an early method of diagnosing and managing patients with lymphadenopathy because of its simplicity, low rate of complications, reduced trauma, and the early availability of results.

Therefore, to improve the diagnosis rate of cytology, it is essential to properly perform FNA, accurately determine clinical findings, and perform precise diagnoses. If the diagnosis is unclear, a parallel histological examination should be performed.

## Figures and Tables

**Table 1:** Clinical characteristics of 432 patients with lymphadenopathy.

	Characteristics	No. of Patients (%)
Gender	Male	247 (57)
	Female	185 (43)
Age (mean age 58.2 years)	<30 years	34 (8)
(range 8–89 years)	30–50 years	96 (22)
	>50 years	302 (70)
Lymph node size (mean size 1.9 cm)	<2.0 cm	265 (61)
(range 0.2–5.0 cm)	2.0–3.0 cm	120 (28)
	>3.0 cm	47 (11)
Lesion location	Cervical	257 (59)
	Supraclavicular	59 (14)
	Mediastinal	86 (20)
	Axillary	22 (5)
	Inguinal	8 (20)

No.: number.

**Table 2:** Comparison of initial cytologic and histologic diagnoses.

Cytologic Diagnosis (*n* = 432)	Histologic Diagnosis (*n* = 432)				
	TIFD (39 ^‡^)	Benign (155)	Metastasis (199)	NHL (35)	HL (4)
Inadequate (15 ^†^)	3	7	5 ^†^	0	0
Benign (155)	24	119	7 ^†^	5 **	0
Atypical (39)	5	16	12	6	0
Suspicious (7)	0	3 ^‡,^*	3	1	0
Metastasis (192)	7 ^‡^	10 ^‡,^*	172	3	0
NHL (20)	0	0	0	20	0
HL (4)	0	0	0	0	4

Inadequate: inadequate/non-diagnostic; TIFD: tissue insufficient for diagnosis; NHL: non-Hodgkin lymphoma; HL: Hodgkin lymphoma. ^†^: sampling errors of FNAC; ^‡^: sampling errors of biopsy. *: overdiagnosed on cytological diagnoses, **: underdiagnosed on cytological diagnoses.

**Table 3:** Distribution of primary sites of the metastatic tumor in lymph nodes.

Primary Site	No. of Cases (*n* = 172)	Histologic Diagnoses	No. of Cases (*n* = 172)	Gender (M:F)	Age (Range)
Lung	58	Adenocarcinoma	26	46:12	43–82
		Squamous cell ca.	19		
		Small cell carcinoma	10		
		Large cell carcinoma	1		
		Mucoepidermoid ca.	1		
		LCNEC	1		
Thyroid gland	50	Papillary thyroid ca.	48	11:39	24–75
		Medullary ca.	2		
Breast	18	Ductal ca.	17	0:18	35–65
		Mucinous ca.	1		
Oral cavity	9	Squamous cell ca.	9	5:4	36–77
Skin	5	Malignant melanoma	4	1:4	48–75
		Squamous cell ca.	1		
Tonsil	5	Squamous cell ca.	3	5:0	38–52
		Adenocarcinoma	2		
Pharynx	4	Squamous cell ca.	2	4:0	48–79
		Adenosquamous ca.	1		
		Undifferentiated ca.	1		
Esophagus	3	Squamous cell ca.	3	3:0	60–68
Ovary	3	Serous ca.	3	0:3	53–76
Bladder	3	Urothelial ca.	3	2:1	60–70
Salivary gland	2	Salivary duct ca.	1	2:0	39–56
		Undifferentiated ca.	1		
Larynx	2	Squamous cell ca.	2	2:0	65–71
Stomach	2	Adenocarcinoma	1	1:1	58–70
		Squamous cell ca.	1		
Rectum	2	Adenocarcinoma	2	1:1	73–76
Liver	1	Hepatocellular ca.	1	0:1	48
Kidney	1	Renal cell ca.	1	1:0	64
Cervix	1	Squamous cell ca.	1	0:1	56
Unknown origin	3	Squamous cell ca.	1	2:1	48–68
		Carcinoma	2		

No.: number, M: male, F: female, Ca.: carcinoma, LCNEC: large cell neuroendocrine carcinoma.

**Table 4:** Histologic diagnoses of FNAC sampling error cases.

Cytologic Diagnosis	No. of Cases (*n* = 22)	Histologic Diagnosis	No. of Cases (*n* = 22)
		TIFD	3
		Benign	7
Inadequate/non-diagnostic	15	Ductal carcinoma	2
		Adenocarcinoma	1
		Squamous cell carcinoma	1
		Papillary thyroid carcinoma	1
		Adenocarcinoma	3
Benign	7	Small cell carcinoma	2
		Squamous cell carcinoma	1
		Papillary thyroid carcinoma	1

No.: number, TIFD: tissue insufficient for diagnosis.

**Table 5:** Cytologic diagnoses of tissue sampling error cases.

Histologic Diagnosis	No. of Cases (*n* = 49)	Cytologic Diagnosis	No. of Cases (*n* = 49)
		Inadequate/non-diagnostic	3
		Benign	24
		Atypical cells	5
TIFD	39	Adenocarcinoma	3
		Small cell carcinoma	2
		Squamous cell carcinoma	1
		Malignant melanoma	1
		Small cell carcinoma	3
		Squamous cell carcinoma	3
Benign	10	Adenocarcinoma	2
		Papillary thyroid carcinoma	1
		Malignant melanoma	1

No.: number, TIFD: tissue insufficient for diagnosis.

**Table 6:** False positive and false negative results of lymph node FNAC.

Cytologic Diagnosis	Histologic Diagnosis	FP/FN (*n* = 8)
Suggestive of malignancy	Foreign body granuloma	FP (*n* = 1)
Suspicious for lymphoma	Reactive hyperplasia	FP (*n* = 1)
Meta. papillary thyroid ca.	Reactive hyperplasia	FP (*n* = 1)
Reactive hyperplasia	Follicular lymphoma, grade 1	FN (*n* = 1)
Reactive hyperplasia	Mantle cell lymphoma	FN (*n* = 1)
Reactive hyperplasia	EBV-positive diffuse large B-cell lymphoma	FN (*n* = 1)
Reactive hyperplasia	Diffuse large B-cell lymphoma	FN (*n* = 1)
Reactive hyperplasia	Diffuse large B-cell lymphoma	FN (*n* = 1)

Meta.: metastatic; Ca.: carcinoma, FP: false positive, FN: false negative.

**Table 7:** Analysis of diagnostic accuracy of FNAC.

Analysis	Group I	Group II
	Malignant + Suspicious (%)	Malignant + Suspicious + Atypical (%)
Sensitivity	97.8	96.0
Specificity	97.5	94.9
Positive predictive value (PPV)	98.7	97.2
Negative predictive values (NPV)	96.0	92.9
Accuracy	97.7	95.6
Under-diagnosis rate	1.3	2.3
Over-diagnosis rate	0.8	1.6
Sampling error rate	5.1	5.1

## Data Availability

Not applicable.
